# Comparison of Germline versus Somatic *BAP1* Mutations for Risk of Metastasis in Uveal Melanoma

**DOI:** 10.1186/s12885-018-5079-x

**Published:** 2018-11-26

**Authors:** K. G. Ewens, E. Lalonde, J. Richards-Yutz, C. L. Shields, A. Ganguly

**Affiliations:** 1 Department of Genetics, Perelman School of Medicine, University of Pennsylvania, 415 Curie Blvd, Philadelphia, Pennsylvania 19104-6145 United States; 2 Oncology Services, Wills Eye Hospital, Thomas Jefferson University, 840 Walnut St, Suite #1440, Philadelphia, Pennsylvania 19107 United States

**Keywords:** uveal melanoma, ocular/eye cancer, *BAP1*, germline mutations, somatic mutations

## Abstract

**Background:**

Germline mutations in *BAP1* have been associated with *BAP1*-Tumor Predisposition Syndrome (*BAP1*-TPDS), a predisposition to multiple tumors within a family that includes uveal melanoma (UM), cutaneous melanoma, malignant mesothelioma and renal cell carcinoma. Alternatively, somatic mutations in *BAP1* in UM have been associated with high risk for metastasis. In this study, we compare the risk of metastasis in UM that carry germline versus somatic *BAP1* mutations and mutation-negative tumors.

**Methods:**

DNA extracted from 142 UM and matched blood samples was sequenced using Sanger or next generation sequencing to identify *BAP1* gene mutations.

**Results:**

Eleven of 142 UM (8%) carried germline *BAP1* mutations, 43 (30%) had somatic mutations, and 88 (62%) were mutation-negative. All BAP1 mutations identified in blood samples were also present in the matched UM. There were 52 unique mutations in 54 tumors. All were pathogenic or likely pathogenic.

A comparison of tumors carrying somatic vs. germline mutations, or no mutations, showed a higher frequency of metastasis in tumors carrying somatic mutations: 74% vs. 36%, *P*=0.03 and 74% vs. 26% *P*<0.001, respectively. Tumors with a somatic mutation compared to mutation-negative had an older age of diagnosis of (61.8 vs. 52.2 years, *P*=0.002), and shorter time to metastasis (16 vs. 26 months, *P*=0.04). Kaplan-Meier analysis further showed that tumors with somatic (vs. germline) mutations demonstrated a greater metastatic risk (*P*=0.03). Cox multivariate analysis showed in addition to chromosome-3 monosomy and larger tumor diameter, the presence of *BAP1* somatic, but not germline mutations, was significantly associated with risk of metastasis(*P*=0.02).

Personal or family history of *BAP1*-TPDS was available for 79 of the cases. All eight cases with germline mutations reported a history of *BAP1*-TPDS, which was significantly greater than what was observed in cases with somatic mutations (10 of 23, *P*=0.009) or mutation-negative cases (11 of 48, *P*<0.001).

**Conclusions:**

Defining germline vs. somatic nature of *BAP1* mutations in UM can inform the individual about both the risk of metastasis, and the time to metastasis, which are critically important outcomes for the individual. This information can also change the cascade screening and surveillance of family members.

**Electronic supplementary material:**

The online version of this article (10.1186/s12885-018-5079-x) contains supplementary material, which is available to authorized users.

## Background

The *BRCA1-associated protein 1 (BAP1)* gene is located on chromosome 3p21 and encodes a nuclear localized, ubiquitin carboxy-terminal hydrolase tumor suppressor protein [1, 2]. BAP1 has been shown to bind to the BRCA1 protein enhancing BRCA1-mediated tumor suppression, and is involved in various biological processes including DNA damage response, regulation of the cell cycle and cell growth [2, 3]. Germline *BAP1* mutations have been associated with hereditary predisposition to multiple different cancers that include uveal and cutaneous melanoma, malignant mesothelioma on exposure to asbestos, renal cell carcinoma and other cancer types, such as lung adenocarcinoma and meningioma, that are collectively referred to as *BAP1* Tumor Predisposition Syndrome (*BAP1*-TPDS, OMIM #614327) [4, 5]. In vivo studies in melanocytic cells, have shown that *BAP1* is involved in the maintenance of a melanocytic phenotype; the depletion of BAP1 protein levels result in dedifferentiation of cells and the acquisition of a more primitive, stem cell like phenotype [6]. It is well established that the *BAP1* region of chromosome 3p is commonly deleted in several cancers including melanomas, breast and lung cancers, among others [1, 7].

Uveal melanoma (UM) is a rare malignant tumor of the eye with a frequency of 5.1 per million in the United States [8], and is the cancer most commonly associated with the *BAP1*-TPDS [9]. The prognosis for UM is poor in approximately 50% of cases primarily due to metastasis to the liver within a short time [10, 11]. Factors such as large tumor size, location and chromosome 3 monosomy [12–18], as well as a specific 12-gene expression pattern [19–21] have all been associated with an increased risk of metastasis. In addition, the presence of inactivating somatic [22–29] or germline [30] *BAP1* mutations often in conjunction with chromosome 3 monosomy, loss of *BAP1* expression, or lack of immunohistochemical staining, have all been associated with metastasizing UM.

Germline *BAP1* mutations have been identified in approximately 2-5% of UM unselected for the presence of any high risk of hereditary cancers [30–33]. These studies reported that metastasis occurred more frequently in UM carrying germline mutations in the blood compared to *BAP1* mutation-negative controls, but the differences were significant only in one study [30], possibly due to the small number of tumors with germline mutations. In studies where DNA from both tumor tissue and blood was sequenced to rule out the presence of germline mutations, the frequency of somatic *BAP1* mutations was considerably higher than that observed in tumors with germline mutations, approaching 50% [23, 27, 34]. In other studies where somatic mutations were detected by BAP1 expression assays and/or negative immunohistochemical staining methods, as well as sequencing, the lack of BAP1 expression was also significantly associated with the risk of metastasis [22–29]. While these studies show a strong association between somatic *BAP1* mutations and metastasis, the effect of germline changes remains unclear.

Most studies of *BAP1* mutation status in UM have been carried out using enucleated UM which may not be representative of how UM are currently treated, especially smaller tumors that are treated with globe sparing procedures. In this study of *BAP1* mutations in UM, the presence of germline or somatic mutations was determined using sequence analysis of DNA extracted from biopsies of both enucleated globes and fine needle aspirations (FNA) and from matched blood samples. Demographic and clinical characteristics of the tumors and the frequency of metastasis were compared among tumors with germline or somatic *BAP1* mutations and mutation-negative UM.

## Methods

### UM cases

A total of 142 UM cases managed by the Ocular Oncology Service at Wills Eye Hospital, Thomas Jefferson University, Philadelphia, PA, USA between 1998 and 2016 were evaluated in this study of somatic and germline *BAP1* mutations. Twenty-three tumor samples were obtained by solid open biopsy of enucleated UM and 119 by fine needle aspirate (FNA) biopsies. Matched peripheral blood samples were obtained from all 142 individuals. Data on demographic and clinical characteristics, metastatic status, location of metastasis and relevant follow-up times were obtained by retrospective chart reviews. Written informed consent was obtained for all individuals at the time of chromosome testing of the tumor DNA. This research was approved by the Institutional Review Board of the University of Pennsylvania, and is in accordance with the Declaration of Helsinki.

The 142 UM were initially collected from two cohorts. Cohort 1 cases (N=90) were chosen from a series of tumors diagnosed between 1998 and 2013 based on availability of matched tumor and blood samples. Cohort 2 consisted of 52 cases diagnosed between 2008 and 2016 for which *BAP1* testing was requested, typically due to a personal and/or family history of cancer. The decision to combine the two sets of tumors into a single cohort for analysis was based on the finding that cohort membership was not a significant variable in either univariate (*P*=0.53) or multivariate (*P*=0.65) Cox regression analysis (Table 1). In addition, there was no significant difference between the two cohorts in the relative number of tumors carrying germline or somatic *BAP1* mutations and mutation*-*negative tumors (*P*= 0.76), or in the number of cases with personal or family history of *BAP1*-TPDS cancers (P=0.35, see below and Additional file 1: Table S1). There was a significant difference in the time to metastasis (P=0.01) and in the number of metastases (P=0.003) between the two cohorts, possibly due to a longer follow-up time for the cohort 1 tumors (median=85 months, range 6-191) compared to 24 months (range 4-77 months, *P*<0.001, Additional file 1: Table S1) for cohort 2. However, taking both metastasis and follow-up time into account in a Kaplan-Meier analysis, there was no significant association between cohort membership and metastasis (*P*=0.52, Additional file 1: Figure S1).

**Table 1:** Demographic and tumor characteristics for 142 UM and assessment of their association with metastasis evaluated by Cox univariate regression for each of the nine variables alone, and multivariate regression that included all variables described in the table

Variables	All tumors *N*=142 (frequency)	Univariate regression			Multivariate regression		
		*P*-value	Hazard ratio	95% confidence intervals	*P*-value	Hazard ratio	95% confidence intervals
Cohort							
1	90 (0.63)		reference			reference	
2	52 (0.37)	0.53	0.82	0.44-1.53	0.65	1.18	0.57-2.48
Source of biopsied sample^a^							
FNA, N=119 (0.84)			reference			reference	
BAP1 mutation negative	73 (0.61)						
BAP1 mutation positive	46 (0.39)						
Enucleated tumor, N= 23 (0.16)		**0.01**	2.12	1.19-3.76	0.08	2.04	0.92-4.52
BAP1 mutation negative	15 (0.65)						
BAP1 mutation positive	8 (0.35)						
Age (years)		**0.04**	1.02	1.00-1.04	**0.03**	1.02	1.00-1.05
Median	56.7						
Mean±SD	55.4±14.7						
Range	14-88						
Sex							
Male	77 (0.54)		reference			reference	
Female	65 (0.46)	0.24	0.73	0.43-1.24	0.73	0.90	0.50-1.63
Chromosome 3							
Disomy	63 (0.44)		reference			reference	
Monosomy, partial monosomy (N=2), mosaic (N=5)	79 (0.56)	**<0.001**	4.64	2.46-8.78	**0.008**	2.99	1.33-6.72
Tumor diameter (mm)		**<0.001**	1.25	1.17-1.35	**<0.001**	1.20	1.10-1.31
Median	12.0						
Mean±SD	12.5±4.1						
Range	5.0-22.0						
Tumor thickness (mm)		**<0.001**	1.22	1.13-1.31	0.61	0.97	0.87-1.09
Median	5.7						
Mean±SD	6.2±3.3,						
Range	1.0-16.5						
Ciliary body involvement							
Absent	110 (0.78)	**<0.001**	reference			reference	
Present	32 (0.22)		3.05	1.79-5.20	0.15	1.54	0.85-2.80
*BAP1*							
Negative	88 (0.62)		reference			reference	
Somatic	43 (0.30)	**<0.001**	4.81	2.79-8.28	**0.02**	2.20	1.13-4.30
Germline	11 (0.08)	0.33	1.70	0.59-4.91	0.81	0.87	0.28-2.72

^a^There was no significant difference in the number of *BAP1* mutation negative tumors and those carrying germline or somatic mutations UM biopsies from FNA samples compared to enucleated tumors (*P*=0.79, Fisher Exact test)

Figures is bold indicate significant *P*-values <0.05

A personal or family history of *BAP1*-TPDS syndrome, defined following the guidelines found in OMIM #614327 [4] and Pilarski et al [5], was available for a subset of the UM cases. Individuals having at least two *BAP1*-TPDS tumors (UM, cutaneous melanoma, malignant mesothelioma, or renal cell carcinoma) themselves, or one in the UM case plus at least one in a first or second degree relative were considered as indicative of *BAP1*-TPDS syndrome. Cases with non-specific cancer diagnoses were not included as *BAP1*-TPDS syndromic tumors,

### *BAP1* mutation screening and chromosome 3 copy number analysis

Genomic DNA was extracted from peripheral blood lymphocytes using the Gentra Puregene Blood Kit (Cat No.158489, Qiagen) following the manufacturer’s protocol, and from tumor samples as described previously [15, 35]. Sanger sequencing of DNA from matched peripheral blood lymphocytes was used to determine the presence of a germline mutation as noted by Abdel-Rahman et al [36]. DNA from enucleated tumor or FNA biopsies was sequenced to identify *BAP1* mutations using either Sanger or next generation sequencing of all coding exons and adjacent intronic regions [37, 38]. Sequence alignment and variant calling was performed as described previously [37]. The 2015 ACMG guidelines were followed in determining the degree pathogenicity of all sequence variants [39].

Chromosome 3 copy number was determined by microsatellite analysis (*N*=51) or whole genome SNP array (Affymetrix Human 100K, SNP-5.0 or SNP-6.0 and Cytoscan HD genotyping arrays, Affymetrix, Santa Clara CA) [15, 35]. Tumors were categorized as either disomy-3 (*N*=63) or monosomy (*N*=79) that included 72 tumors with complete monosomy-3, two with partial monosomy and five with a mosaic monosomy 3 indicating tumor heterogeneity of cells with chromosome-3 disomy and monosomy.

### Statistical analyses

Continuous variables (age, tumor diameter and thickness) were compared using a Mann-Whitney U-test, and discrete variables (source of tumor sample, cohort 1 or 2, metastasis status, sex, tumor location and chromosome 3 copy number) using Fisher Exact or chi-square tests (vassarstats.net). Cox univariate and multivariate proportional hazard regression was used to determine the association of clinical and tumor characteristics with metastasis. The Kaplan-Meier method was used to generate metastasis-free survival curves that were compared using a log-rank test. SPSS 24 (IBM, New York, NY) was used for survival analysis procedures. All tests were two-tailed and P-values less than 0.05 were considered statistically significant.

## Results

### UM individuals and tumor characteristics

Of the 142 UM characterized in this study, 54 (38%) carried a *BAP1* gene mutation. Eleven (8%) of the mutations were germline and 43 (30%) were somatic. The remaining 88 (62%) were *BAP1* mutation-negative. The eleven germline mutations were all retained in the matched UM samples. Thus, there were no cases where a germline mutation was lost in tumors, with or without monosomy-3.

Demographic and tumor characteristics for the 142 UM cases are shown in Table 1. The median age of all individuals was 56.7 years (range=14-88), and 77 (54%) were males. Overall, 63 (44%) of the tumors carried two copies of chromosome 3 (disomy), and 79 (56%) carried one copy of chromosome 3 (monosomy), partial monosomy or mosaic. The median tumor diameter and thickness was 12 mm (range=5.0-22.0) and 5.7 mm (range 1.0-16.5), respectively. The majority of the tumors had no ciliary body involvement (*N*=110, 78%). Most tumors were located only in the choroid (*N*=109, 77%), 26 (18%) were in the choroid plus ciliary body, three (2%) in the ciliary body, one (1%) in the iris, two (2%) in the iris plus ciliary body, and one (1%) in the iris plus ciliary body and choroid. Fifty-nine tumors (42%) metastasized within a median time of 19 months (range=0-107) while 83 (58%) did not metastasize during the observed follow-up period (median=56 months, range=4-191 months, Table 2). Thirty-six of 54 tumors with *BAP1* mutations metastasized (67%) compared to only 23 of 88 (26%) mutation-negative tumors (*P*<0.001) within 2-107 months.

**Table 2:** Description of demographic and tumor variables with pairwise comparisons for 11 tumors with germline BAP1 mutations, 43 with *BAP1* somatic mutations and 88 with no *BAP1* mutations

Variables	*BAP1* germline	*BAP1* somatic	*BAP1* negative	Pairwise comparisons (*P*-value)		
	*N*=11 (0.08) (frequency)	*N*=43 (0.30) (frequency)	*N*=88 (0.62) (frequency)	Germline/Somatic	Germline/Negative	Somatic/Negative
Metastases						
Yes N=59 (0.42)	4 (0.36)	32 (0.74)	23 (0.26)	**0.03** ^a^	0.72^a^	**<0.001** ^a^
No N=83 (0.58)	7 (0.64)	11 (0.26)	65 (0.74)			
Time to metastasis (months) (*N*=59)	N=4 (0.07)	N=32 (0.54)	N=23 (0.38)	0.07^b^	0.50^b^	**0.04** ^c^
Median (19.0 months)	37.5	16.0	26.0			
Mean±SD (26.4±20.9)	35.0±15.8	23±22.5	29.7±19.2			
Range (0-107)	16-49	2-45,90,107^d^	0-84			
Follow-up time (months) in tumors with no metastasis (*N*=83)	N=7 (0.08)	N=11 (0.13)	N=65 (0.78)	0.59^c^	0.25^c^	0.33^c^
Median (56.0 months)	24	54.0	58.0			
Mean±SD (59.8±39.0)	43.7±39.3	49.9±30.0	63.2±40.1			
Range (4-191)	4-109	8-90	5-191			
Cohort						
1	6 (0.54)	27 (0.63)	57 (0.65)	0.73^a^	0.74^a^	0.85^a^
2	5 (0.46)	16 (0.37)	31 (0.35)			
Source of biopsied sample						
FNA	10 (0.91)	36 (0.84)	73 (0.83)	0.68^a^	0.69^a^	1.00^a^
Enucleated tumor	1 (0.09)	7 (0.16)	15 (0.17)			
*BAP1*-TPDS personal or family history (*N*=79 reports)^e^						
Yes, syndromic tumors present (*N*=29, 0.37)	8 (1.00)	10 (0.44)	11 (0.23)	**0.009** ^a^	**<0.001** ^a^	0.10
No syndromic tumors (N=50, 0.63)	0	13 (0.56)	37 (0.77)			
Age (years)						
Median	59.0	61.8	52.2	0.07^c^	0.86^c^	**0.002** ^c^
Mean±SD	51.9±13.6	61.2±12.8	53.1±15.0,			
Range	22-67	28-88	14-84			
Sex						
Male	7 (0.64)	25 (0.58)	45 (0.51)	1.00^a^	0.53^a^	0.46^a^
Female	4 (0.36)	18 (0.42)	43 (0.49)			
Chromosome 3						
Disomy	1 (0.09)	3 (0.07)	59 (0.67)	1.0^a^	**<0.001** ^a^	**<0.001** ^a^
Monosomy, partial monosomy (*N*=2), mosaic (*N*=5)	10 (0.91)	40 (0.93)	26 (0.33)			
Tumor diameter (mm)						
Median	14.0	14.0	11.5	0.70^c^	0.23^c^	**0.003** ^c^
Mean±SD	13.4±4.4	13.9±3.9	11.7±4.0,			
Range	8.0-20.0	6.0-21.5	5.0-22.0			
Tumor thickness (mm)						
Median	6.6	7.0	5.0	0.41^c^	0.51^c^	**0.002** ^c^
Mean±SD	6.5±3.6	7.5±3.5	5.6±2.9,			
Range	1.5-12.3	2.0-16.5	1.0-13.1			
Ciliary body involvement						
Absent	8 (0.73)	28 (0.65)	74 (0.84)	0.73^a^	0.40^a^	**0.02** ^a^
Present	3 (0.27)	15 (0.35)	14 (0.14)			

^a^Association test performed using two-tailed Fisher Exact or Chi-square tests

^b^Reported P-values are calculated from a normal approximation of the Mann Whitney test statistic

^c^Tests of means of quantitative variables performed using Mann-Whitney U tests

^d^The time for metastasis for 30 of the tumors with somatic mutations was 2-45 months. The time to metastasis for the remaining two tumors was 90 and 107 months

^e^*BAP1*-TPDS, *BAP1*-tumor predisposition syndrome as defined in OMIM #614327 [4] and Pilarski et al [5]

Figures is bold indicate significant P-values <0.05

*BAP1* status was determined for 119 (84%) FNA biopsies and 23 enucleated tumors (Table 1). There was a significant difference in the number of metastases depending on the source of the biopsied sample: 43 of 119 (36%) tumors biopsied from FNA metastasized compared to 16 of 23 from enucleated globes (70%, *P*=0.006). The source of the biopsied sample was also significantly associated with metastasis in a Cox univariate (*P*=0.01), but not in a multivariate analysis (0.08, Table 1). This is not surprising, however, given that some of the classic prognostic factors for UM metastasis, larger tumor diameter and thickness and tumor location which warrant enucleation, were significantly different between tumors sampled from FNAs or enucleated tumor samples (*P*=0.002, >0.001 and 0.02, respectively (data not shown). Importantly, there was no difference in the number of tumors carrying a germline or somatic mutation or mutation-negative tumors in enucleated tumors compared to FNA samples (*P*=0.79, Table 1 footnote^a^).

In a Cox univariate analysis, the presence of a somatic, but not a germline *BAP1* mutation (hazard ratio (HR) =4.81, *P*<0.0001; HR=1.7, *P*=0.33, respectively, Table 1) was significantly associated with metastasis. Other tumor variables that were significant in the univariate regression analysis included tumor source (FNA or enucleated tumor, HR= 2.12; *P*=0.01), age HR=1.02, *P*=0.04), chromosome 3 monosomy, larger tumor diameter and thickness, and ciliary body involvement (HR=4.64, 1.25 and 1.22, respectively; all *P*≤0.001). Considering all variables in a multivariate analysis, the presence of a somatic *BAP1* mutation (HR=2.20, P=0.02), age (HR=1.02, *P*=0.03) tumor diameter (HR=1.20, *P*<0.001), and chromosome 3 monosomy (HR=2.99, *P*=0.008) remained significant, while the presence of a germline mutation was not significant (HR=0.87, *P*=0.81).

### *BAP1* Gene Mutations

Fifty-two unique *BAP1* mutations were identified in 54 tumors: 43 tumors (30%) carried somatic mutations, while 11 tumors (8%) carried germline mutations (Table 3). All mutations were defined as pathogenic or likely pathogenic. These included seven (13%) missense and 15 (28%) splice-site mutations, three (6%) in-frame deletions and 29 mutations causing premature truncation, including one large deletion encompassing exons 15-17 and the intervening introns. There was no significant difference in the frequencies of these categories of mutations found in UM with germline compared to somatic mutations (*P*=1.0), nor in tumors that metastasized compared to those that did not (*P*=0.53). Thirty-six (67%) of the mutations were in the catalytic UCH domain. One splice-site mutation, c.438-1A>G was present as a germline mutation in one tumor (UM-23) and as a somatic mutation in two tumors (UM-11 and UM-27). Thirty-three of these mutations were previously described in UM by Ewens et al [37] and 13 have been identified in UM by others (as noted in Table 3). Although the number of germline mutations was small (*N*=11), the locations of these were spread throughout the gene, similar to the distribution of somatic mutations (Table 3). Therefore, no new information was inferred from the location, or the associated functional domains, that would make the germline mutations have unique characteristic compared to somatic mutations.

**Table 3:** Fifty-two unique germline and somatic *BAP1* mutations identified in 54 UM

ID	BAP1 mutation	Metastases	chr 3	DNA change		Protein change	Predictred effect	Predicted pathogenic effect^a^	Previously reported mutations in UM
UM_44	germline	yes	monosomy	exon 1	c.3G>A	p.Met1Ile	misense: start site lost	LP	[36]
UM_76	somatic	yes	monosomy	intron 1	c.38-1G>C	p.?	splicing	P	[36]
UM_9	somatic	yes	monosomy	exon 2	c.40_52del13	p.Leu14SerfsTer54	truncating	P	[36]
UM_1136	germline	no	monosomy	exon 2	c.58_59insTG	p.Glu20ValfsTer53	truncating	P	
UM_113	somatic	yes	monosomy	intron 2	c.67+1G>A	p.?	splicing	P	[27, 36]
UM_1123	somatic	yes	monosomy	intron 2	c.67+2dupT	p.?	splicing	P	
UM_1080	somatic	no	monosomy	intron 2/exon 3	c.68-12_75del20	p.?	splicing	P	
UM_802	somatic	yes	monosomy	intron 2/exon 3	c.68-16_69del18	p.?	splicing	P	[36]
UM_584	germline	no	monosomy	exon 3	c.79delG	p.Val27CysfsTer45	truncating	P	
UM_17	somatic	yes	monosomy	exon 3	c.82C>T	p.Gln28Ter	truncating	P	[36]
UM_1207	somatic	no	monosomy	exon 3	c.91_93delGAG	p.Glu31del	in-frame deletion	LP	
UM_1133	germline	no	disomy^b^	intron 3	c.122+1G>C	p.?	splicing	P	
UM_109	germline	yes	monosomy	intron 3	c.122+1G>T	p.?	splicing	P	[36]
UM_75	somatic	no	monosomy	exon 4	c.125_145del21	p.Pro42_Phe48del	in-frame deletion	LP	[36]
UM_88	somatic	yes	monosomy	exon 4	c.145delC	p.Leu49CysfsTer23	truncating	P	[36]
UM_877	somatic	yes	monosomy	exon 4	c.165_180del16	p.Arg57SerfsTer10	truncating	P	
UM_13	somatic	yes	disomy^b^	exon 4	c.178C>T	p.Arg60Ter	truncating	P	[36, 45]
UM_62	somatic	yes	monosomy	exon 4	c.202_227del26	p.Asp68CysfsTer3	truncating	P	[36]
UM_780	somatic	yes	monosomy	exon 4/intron 4	c.234del96	p.?	splicing	P	
UM_119	somatic	yes	monosomy	exon 4	c.253C>T	p.Gln85Ter	truncating	P	[36]
UM_106	somatic	yes	monosomy	exon 4	c.254A>C	p.Gln85Pro	missense	P	[36]
UM_1126	somatic	no	monosomy	exon 5	c.295_312del18	p.Val99_Ser104del	in-frame deletion	LP	
UM_1046	somatic	no	monosomy	exon5/intron5	c.370_375+12del18	p.?	splicing	P	
UM_1208	somatic	yes	monosomy	intron 5/exon 6	c.376-20_383del28	p.?	splicing	P	
UM_56	somatic	yes	monosomy	exon 6	c.422A>G	p.His141Arg	missense	LP	[36, 46]
UM_23	germline	yes	monosomy	intron 6	c.438-2A>G	p.?	splicing	P	[36]
UM_11	somatic	no	disomy^b^	intron 6	c.438-2A>G	p.?	splicing	P	
UM_27	somatic	yes	monosomy	intron 6	c.438-2A>G	p.?	splicing	P	
UM_58	germline	no	monosomy	exon 7	c.458_459delCT	p.Pro153ArgfsTer7	truncating	P	[9, 36]
UM_114	somatic	yes	monosomy	exon 7	c.497_509del13	p.Glu166ValfsTer17	truncating	P	[36]
UM_51	somatic	no	monosomy	exon 7	c.506A>C	p.His169Pro	missense	P	[36]
UM_1118	somatic	no	monosomy	exon 7	c.524C>G	p.Pro175Arg	missense	LP	[27]
UM_1086	somatic	yes	monosomy	intron 7/exon 8	c.581-2_591del13	p.?	splicing	P	
UM_60	somatic	yes	monosomy	exon 8	c.588G>A	p.Trp196Ter	truncating	P	[23, 47]
UM_1334	germline	no	mosaic	exon 8	c.619delC	p.Arg207GlyfsTer24	truncating	P	
UM_105	somatic	yes	monosomy	intron 8	c.659+1G>A	p.?	splicing	P	[27, 36]
UM_35	somatic	yes	monosomy	exon 9	c.723T>A	p.Tyr241Ter	truncating	P	[36, 48:c.723T>C]
UM_107	somatic	yes	monosomy	exon 9	c.781C>T	p.Gln261Ter	truncating	P	[36, 49] rs772448753^c^
UM_46	somatic	yes	monosomy	exon 10	c.904_905insT	p.Pro302LeufsTer5	truncating	P	[36]
UM_1029	somatic	yes	monosomy	exon 12	c.1134_1143del10_insAA	p.Ala379ArgfsTer16	truncating	P	
UM_115	somatic	yes	monosomy	exon 12	c.1153C>T	p.Arg385Ter	truncating	P	[32, 36, 44, 50]
UM_55	somatic	no	monosomy	exon 12	c.1175_1182delAGCAGTAC	p.Gln392LeufsTer3	truncating	P	[36]
UM_863	somatic	no	monosomy	exon 12	c.1192G>T	p.Glu398Ter	truncating	P	
UM_950	germline	yes	mosaic	exon 12	c.1203dupT	p.Glu402Ter	truncating	P	
UM_69	somatic	yes	monosomy	exon 12	c.1217_1220delAGGA	p.Glu406ValfsTer23	truncating	P	[36]
UM_1333	germline	no	mosaic	exon 13	c.1695dupT	p.Glu566Ter	truncating	P	
UM_48	somatic	no	monosomy	exon 13	c.1729G>C	p.Glu577Gln	missense	P	[36]
UM_61	somatic	yes	monosomy	exon 14	c.1881C>G	p.Tyr627Ter	truncating	P	[36]
UM_74	germline	no	monosomy	exon 14	c.1882_1885delTCAC	p.Ser628ProfsTer8	truncating	P	[29, 36, 51]
UM_708	somatic	no	disomy^b^	intron 14-3'UTR	c.1890+38_2573del	p.Glu631Ter	truncating (3 exon deletion)	P	
UM_1113	somatic	yes	monosomy	exon 15	c.1926_1951del 26	p.Ile643GlyfsTer12	truncating	P	
UM_104	somatic	yes	monosomy	exon 15	c.1932_1948deldel17	p.Asn645GlnfsTer13	truncating	P	[36]
UM_804	somatic	yes	monosomy	exon 16	c.1986_1989delTGAT	p.Ile662MetfsTer29	truncating	P	[36]
UM_108	somatic	yes	monosomy	exon 16	c.2015A>G	p.Asp672Gly	missense	LP	[23, 36]

^a^Predicted pathogenic effect based on ACMG guidelines [39] P, pathogenic; LP, likely pathogenic

^b^indicates UM with chromosome 3 disomy and carrying a germline or somatic BAP1 mutation

**Population frequency rs772448753: 8.2E-06

### Characterizations of UM with and without *BAP1* mutations

Clinical and tumor characteristics of UM carrying *BAP1* germline or somatic mutations and mutation-negative tumors, as well as pairwise comparisons of the three groups are presented in Table 2. There was no significant difference in the cohort, source of biopsy sample, or sex of the individuals among the three categories of mutations. The age at diagnosis of individuals with somatic mutations was significantly older (61.8 years, range=28-88) than individuals with mutation-negative tumors (median=52.2 years, range=14-84 years, *P*=0.002) and approached significance compared to individuals with a tumor carrying a germline mutation (median=59.0 years, range=22-67, *P*=0.07).

There was a significant difference in the comparison of chromosome 3 monosomy in mutation-negative tumors compared to tumors with either germline or somatic mutations. Only 26 (33%) mutation-negative tumors carried monosomy 3, while ten (91%) tumors with germline mutations and 40 (93%) with somatic mutations were chromosome 3 monosomy (*P*<0.001 in both cases). A comparison of chromosome 3 copy number between tumors with germline vs. somatic mutations (91% vs. 93%) was not significant (*P*=1.0, Table 2). There were four tumors with *BAP1* mutations and chromosome 3 disomy (marked with superscript b in Table 3). These would suggest that chromosome 3 loss was not a necessary consequence of *BAP1* loss. The one germline mutation was a splice-site mutation, intron 3, c.122+1G>C. The three somatic mutations included one splice-site mutation (intron 6, c.438-2A>G) and two truncating mutations: exon 4, c.178C>T (p.Arg60X) and a large deletion of exons 15-17 and the intervening introns. The tumor carrying the exon 4, c.178C>T (p.Arg60X) truncating mutation metastasized at 11 months, but the other three tumors had not metastasized within 24 months (c.122+1G>C) or within at least 65 months (c.438-2A>G and the large three exon deletion).

Tumors with *BAP1* somatic mutations had a significantly larger median diameter (14.0 mm, range=6.0-21.5) and thickness (7.0 mm, range=2.0-16.5) compared to mutation negative tumors (median diameter=11.5 mm, range=5.0-22.0; thickness=5.0 mm, range=0.0-13.1, *P*=0.003 and P=0.002 respectively, Table 2). However, there was no significant difference in the tumor diameter or thickness between *BAP1* germline tumors and tumors with either somatic mutations (*P*=0.70 and *P*=0.41, respectively), or *BAP1*-negative tumors (P=0.23 and *P*=0.51, respectively). Table 2 shows that there was also a significant difference in ciliary body involvement of tumors carrying a somatic mutation, where the tumor was more likely to have ciliary body involvement (33%) compared to *BAP1*-negative tumors (14%, P=0.04), but not compared with those with a germline mutation (27%, *P*=1.0).

**Fig. 1:**
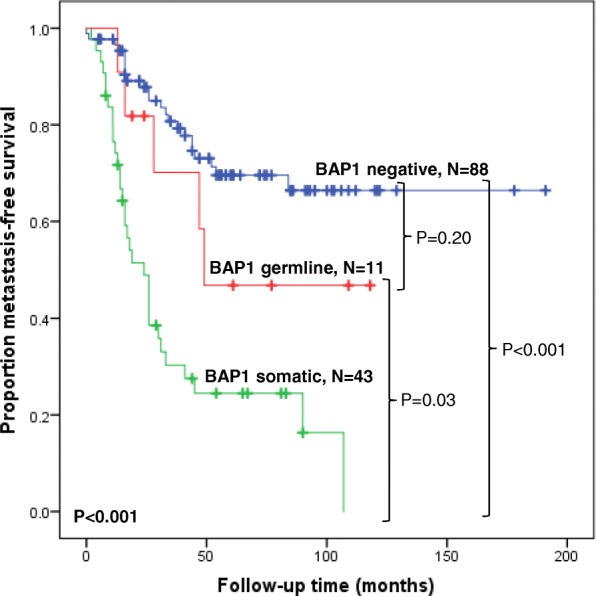
Kaplan-Meier curves showing metastasis-free survival following diagnosis for 142 UM stratified by *BAP1* mutation status

Tumors carrying *BAP1* somatic mutations metastasized significantly more often (32 of 43, 74%) compared to those with either *BAP1* germline mutations (4 of 11, 36%, *P*=0.03) or to *BAP1* mutation-negative tumors (23 of 88, 26%, *P*<0.001, Table 2). However, there was no significant difference in the frequency of metastasis between tumors with germline mutations and *BAP1*-negative tumors (*P*=0.72). This finding was corroborated by the multivariate regression analyses shown in Table 1 indicating that somatic, but not germline *BAP1* mutations, were significantly associated with metastasis (*P*=0.03 and 0.70, respectively). Kaplan-Meier analysis (Figure 1) also showed that tumors with a *BAP1* somatic mutation had a significantly poorer metastatic outcome compared to those with germline mutations (P=0.03) or mutation-negative tumors (*P*<0.001), but the difference between tumors with germline mutations and *BAP1* mutation-negative tumors was not significant (P=0.23). The time to metastasis was also significantly shorter in the tumors with somatic mutations (median=16.0 months, range=2-107) compared to *BAP1-negative* tumors (median=26 months, range=0-84 months, *P*=0.04, Table 2).

A positive personal and/or family history of *BAP1*-TPDS tumors was reported for 29 (37%) of the 79 cases that provided information of other cancers in themselves or their families (Table 2). There were eight cases that carried a germline mutation with information on personal or familial cancers; all had a positive history of *BAP1*-TPDS. Comparisons of *BAP1*-TPDS tumors in UM cases with a germline (8, 100%) vs. somatic mutation (10, 44%) or germline vs. mutation-negative (11, 23%) were significant (*P*=0.009 and *P*<0.001, respectively), while a comparison of those with somatic mutations was not significantly different from those with mutation-negative tumors (*P*=0.10)

## Discussion

Inactivating somatic mutations in the *BAP1* gene in UM were first identified and associated with metastatic disease in 2010 [23]. Subsequent to this original publication, there have been numerous studies characterizing *BAP1* somatic [22, 24–29, 40–43] and germline [9, 30, 32, 33] mutations in UM. However, to our knowledge, this is the first report directly comparing risk of metastasis in tumors carrying germline or somatic *BAP1* mutations and mutation-negative tumors. In this study, the presence of somatic mutations was strictly defined as those found in tumors, and not in matched blood samples. A second important difference in this study is that, compared to most other reports where UM tumors analyzed for *BAP1* mutations were comprised primarily of samples biopsied from larger, enucleated tumors, 84% of the samples analyzed in this study were from FNA biopsies. We found no significant difference in the frequency of somatic and germline mutations in biopsies from FNAs compared to enucleated tumors. Since the majority of UM are currently being treated by globe sparing procedures, it was important to determine whether the consequences of carrying a *BAP1* mutation identified in larger enucleated tumors could be applied to smaller UM biopsied by FNA sampling.

It is well established that tumor characteristics such as tumor diameter, ciliary body involvement and chromosome 3 monosomy are all associated with poor prognosis as measured by the development of metastases within 48 months after the primary is treated [12–18]. It has also been shown that that these same variables are significantly associated with *BAP1* mutation status in tumors with somatic or germline mutations [24, 25, 28, 30]. In this study, multivariate regression analysis showed that in addition to these classic tumor variables, the presence of somatic, but not germline, *BAP1* mutations was significantly associated with metastasis (HR=2.20, *P*=0.02; HR=0.87, P=0.81, respectively). Kaplan-Meier analysis further showed that tumors with somatic mutations were associated with the poorest metastatic outcome, while there was no significant difference between tumors carrying a germline mutation and mutation-negative tumors. Furthermore, the time to metastasis was seven months shorter for individuals whose tumors carried somatic mutations compared to mutation-negative tumors (*P*=0.04) suggesting a more aggressive phenotype. Thus, in a previous publication [37] we showed that tumors with any mutation in the *BAP1* gene, in combination with chromosome 3-monosomy, have the highest risk of metastasis (HR=11.5). In this manuscript we show that monosomy 3 plus a somatic mutation in the *BAP1* gene, as opposed to a germline mutation, have a significantly higher risk of metastasis (*P*=0.01, H.R.=2.81) and a shorter time to metastasis compared to the ones with a germline mutation.

Given that somatic *BAP1* mutations appear to have a highly significant association with poor metastatic prognosis, it is surprising that the age of diagnosis of the individuals whose tumors carried somatic mutations was significantly older (median=61.8 years) compared to those with mutation-negative tumors (median=52.2 years, *P*=0.002). Previous studies have also reported that tumors with somatic mutations were diagnosed at least 8 years later than mutation-negative controls [24, 27, 29], but the significance of this observation is not clear.

Among the 54 tumors with *BAP1* mutations, there were four with chromosome 3 disomy. It cannot be ruled out that although these tumors appeared to be disomy-3, small deletions not detectable on the SNP arrays leading to partial monosomy or heterogeneity of the tumor sample could be responsible for this finding. Alternatively, in some cases it is possible the during tumor evolution, *BAP1* mutation on one copy of chromosome 3 could precede the loss of the other copy of chromosome 3. While several studies have found total concordance between the presence *BAP1* mutations and monosomy 3 [23, 27, 40], others using sequencing, gene expression, and/or immunostaining methods have identified *BAP1* mutations in a small number of tumors with disomy 3 [24, 25, 28, 44].

One interesting observation in this study was the presence of *BAP1*-TPDS history in 44% of somatic cases and 23% of mutation negative tumors. The data on *BAP1*-TPDS was based on self-reported personal and family history of cancer. We classified individuals as having *BAP1*-TPDS if they carried at least two relevant tumors in their personal or family history. Since it is self-reported, the information can be imprecise, and there is a possibility for over/under representation of the *BAP1-*TPDS cases as well.

While the presence of germline *BAP1* mutations in cancers associated with *BAP1*-TPSD is well documented, the role of somatic mutations in this syndrome is not well studied. It is possible that the presence of TPDS in our cohort of UM cases that have a somatic *BAP1* mutation or are mutation-negative could be interpreted as evidence for the existence of a second type of the syndrome associated with mutations in a second gene different from *BAP1*.

A major strength of this study is that it includes UM sampled from enucleated tumors and FNAs with both somatic and germline *BAP1* mutations. However, it is limited by the small number of tumors with germline mutations. It is important to confirm these findings in a larger cohort of UM.

## Conclusions

This study identified significant differences in the risk of metastasis for individuals whose tumors carry somatic vs. germline *BAP1* mutations. While overall 36 of 59 (61%) metastasizing tumors carried a *BAP1* mutation, only 7% carried germline mutations, compared to 54% with somatic mutations. From the viewpoint of counseling individuals with UM, there are different implications depending on whether the mutation is classified as germline or somatic. The most relevant information for the individuals with UM is the risk of metastasis, as well as an estimate of metastasis-free survival. The presence of germline mutations can provide the individual with information concerning the risk of development of other cancers themselves or in other family members, while information about the presence of somatic mutations is relevant to their individual risk of developing metastases within a shorter time period. Thus it is important to determine whether a tumor carries a *BAP1* mutation, and if positive, to also evaluate a matched blood sample to establish whether the mutation is germline or somatic. In addition, this information can also change the cascade screening and surveillance of the family members.

## Additional file

Additional file 1: **Table S1.** Comparison of demographic and tumor variables of two cohorts of 142 UM. Description of demographic and tumor variables with pairwise comparisons for 142 divided into two cohorts depending on whether tumors were selected from archived samples for which both tumor and blood samples were available (cohort 1, *N*=90) or tumors for which *BAP1* sequencing was specifically requested (cohort 2, *N*=52). References: 1. OMIM: Tumor predisposition syndrome; TPDS. https://www.omim.org/entry/614327. 2. Pilarski R, Rai K, Cebulla C, Abdel-Rahman M.BAP1 Tumor Predisposition Syndrome. 2016 Oct 13 In: Adam MP, Ardinger HH, Pagon RA, Wallace SE, Bean LJH, Stephens K, Amemiya A, editors. GeneReviews® [Internet]. Seattle (WA): University of Washington, Seattle; 1993-2018. Available from: https://www.ncbi.nlm.nih.gov/books/NBK390611/. **Figure S1.** Kaplan-Meier analysis comparing metastasis-free survival of two cohorts of 142 UM. Kaplan-Meier curves showing metastasis-free survival following treatment for 142 UM stratified by cohort, as described in the legend for Additional file 1: Table S1. (DOCX 81 kb)

## Abbreviations

BAP1: BRCA1-associated protein 1
*BAP1*-TPDS: *BAP1* tumor predisposition syndrome
FNA: Fine needle aspirate
GDL: Genetic Diagnostic Laboratory, University of Pennsylvania, Perelman School of Medicine
UM: Uveal melanoma
vs: Versus
