# Supplementary material for: Comparison of Germline versus Somatic BAP1 Mutations for Risk of Metastasis in Uveal Melanoma
Source: BMC Cancer. 2018 Nov 26;18:1172. doi: 10.1186/s12885-018-5079-x (PMC6260582; doi:10.1186/s12885-018-5079-x)
Supplement: Supplementary file 1 — Table S1. Comparison of demographic and tumor variables of two cohorts of 142 UM. Description of demographic and tumor variables with pairwise comparisons for 142 divided into two cohorts depending on whether tumors were selected from archived samples for which both tumor and blood samples were available (cohort 1, N=90) or tumors for which BAP1 sequencing was specifically requested (cohort 2, N=52). References: 1. OMIM: Tumor predisposition syndrome; TPDS. https://www.omim.org/entry/614327. 2. Pilarski R, Rai K, Cebulla C, Abdel-Rahman M.BAP1 Tumor Predisposition Syndrome. 2016 Oct 13 In: Adam MP, Ardinger HH, Pagon RA, Wallace SE, Bean LJH, Stephens K, Amemiya A, editors. GeneReviews® [Internet]. Seattle (WA): University of Washington, Seattle; 1993-2018. Available from: https://www.ncbi.nlm.nih.gov/books/NBK390611/. Figure S1. Kaplan-Meier analysis comparing metastasis-free survival of two cohorts of 142 UM. Kaplan-Meier curves showing metastasis-free survival following treatment for 142 UM stratified by cohort, as described in the legend for Additional file 1: Table S1. (DOCX 81 kb) [file 12885_2018_5079_MOESM1_ESM.docx]

**Additional file 1**

**Table S1**. Description of demographic and tumor variables with pairwise comparisons for 142 divided into two cohorts depending on whether tumors were selected from archived samples for which both tumor and blood samples were available (cohort 1, N=90) or tumors for which *BAP1* sequencing was specifically requested (cohort 2, N=52).

| Variables | Cohort 1  N=90  (frequency) | Cohort 2  N=52  (frequency) | Pairwise comparisons |
| --- | --- | --- | --- |
| Source of biopsied sample* |  |  |  |
| FNA | 72 (0.80) | 47 (0.90) | 0.16^a^ |
| Enucleated tumor | 18 (0.20) | 5 (0.10) |  |
| *BAP1*-TPDS personal or family history (N=79 reports)^b^ |  |  |  |
| Yes, syndromic tumors present | 9 | 20 | 0.35 |
| No syndromic tumors | 21 | 29 |  |
| Metastasis |  |  | **0.003^a^** |
| No | 44 (0.49) | 39 (0.75) |  |
| Median follow-up time (months) | 85 | 24 | **<0.001^c^** |
| Mean±SD  Range | 86.4±32.5  6-191 | 29.9±18.8  4-77 |  |
| Yes | 46 (0.51) | 13( 0.26) |  |
| Median time to  metastasis (months) | 26 (0.51) | 12 (0.25) | 0.01^c^ |
| Mean±SD  Range | 29.4±21.9  0-107 | 16.0±12.9  2-45 |  |
| *BAP1* mutation |  |  |  |
| Germline | 6 (0.07) | 5 (0.10) | 0.76^a^ |
| Somatic | 27 (0.30) | 16 (0.31) |  |
| Negative | 57 (0.63) | 31 (0.59) |  |
| Sex |  |  |  |
| Male | 56 (0.62) | 21 (0.40) | **0.01^a^** |
| Female | 34 (0.38) | 31 (0.60) |  |
| Age |  |  |  |
| Median  Mean±SD  Range | 60.0  58.5±13.6  22-88 | 52  50.1±15.0  14-82 | **0.001^c^** |
| Tumor diameter (mm) |  |  |  |
| Median  Mean±SD  Range | 12.0  12.4±3.8  5.0-21.0 | 12.0  12.6±4.6  5.0-22.0 | 0.94^c^ |
| Tumor thickness (mm) |  |  |  |
| Median  Mean±SD  Range | 5.8  6.0±3.1  1.0-16.5 | 5.5  6.6±3.6  1.7-15.0 | 0.60^c^ |
| Ciliary body involvement |  |  |  |
| Absent | 66 (0.73) | 44 (0.85) | 0.15^a^ |
| Present | 24 (0.27) | 8 (0.15) |  |
| Chromosome 3 |  |  |  |
| Disomy | 40 (0.44) | 23 (0.44) | 1.0^a^ |
| Monosomy, partial monosomy (N=2), mosaic (N=5) | 50 (0.56) | 29 (0.56) |  |

^a^ Association test performed using two-tailed Fisher Exact or Chi-square tests.

^b^ *BAP1*-TPDS, *BAP1*-tumor predisposition syndrome as defined in OMIM #614327 [1] and Pilarski et al [2].

^c^ Tests of means of quantitative variables performed using Mann-Whitney U tests.

Figures is bold indicate significant P-values <0.05.

**Figure S1**. Kaplan-Meier curves showing metastasis-free survival following treatment for 142 UM stratified

divided into two cohorts depending on whether tumors were selected from archived samples for which both tumor and blood samples were available (cohort 1, N=90) or tumors for which *BAP1* sequencing was specifically requested (cohort 2, N=52).


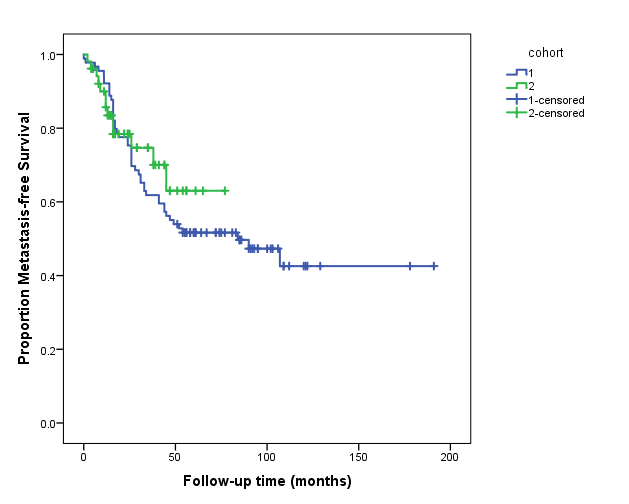


**Cohort 2, N=52**

**Cohort 1, N=90**

P=0.52
